# Clinical and Functional Outcomes of Contemporary Salvage Robotic-Assisted Radical Prostatectomy for Failed Primary Therapy: A Single Institution Review

**DOI:** 10.3390/jcm15218424

**Published:** 2026-10-29

**Authors:** Emily Ye, Randall G. Bissette, Mauricio Vassallo, Maxwell Sandberg, Ashok Hemal, Alejandro R. Rodriguez

**Affiliations:** 1 Wake Forest University School of Medicine, Winston-Salem, NC 27101, USA; 2 Department of Urology, Atrium Health Wake Forest Baptist Medical Center, Winston-Salem, NC 27517, USA

**Keywords:** salvage robotic-assisted radical prostatectomy, recurrent localized prostate cancer, robotic-assisted radical prostatectomy, biochemical recurrence, urinary incontinence, erectile dysfunction

## Abstract

**Background/Objectives**: Salvage radical prostatectomy (SRP) is a potentially curative option for recurrent localized prostate cancer after primary radiation or focal therapy, historically limited by technical complexity and poor functional outcomes. This study characterizes contemporary perioperative, oncologic, and functional outcomes of salvage robotic-assisted radical prostatectomy (sRARP) at a single institution over two decades. **Methods**: We retrospectively reviewed patients undergoing SRP from 01/01/2004 to 06/30/2026, identified via institutional database queries and natural language processing keyword searches with chart confirmation; analysis was restricted to the robotic-assisted subset. Complications within 90 days were graded by the Clavien–Dindo classification. Biochemical recurrence-free survival (bRFS) required freedom from PSA persistence and recurrence (PSA ≥ 0.2 ng/mL, with biochemical recurrence documented in the medical record) and is reported as a proportion. **Results**: Of 4002 records identified, 30 patients met inclusion criteria; 24 (80%) underwent sRARP. Extraprostatic disease was present in 54% (13/24) and positive margins in 43% (10/23). Within 90 days, 43% (10/23) had a complication (13% grade ≥ III); 30% (7/23) had a later complication, and 4% (1/24) required later urinary diversion. At a median follow-up of 32.1 months, bRFS was 39% (9/23), and 33% (8/24) had PSA persistence. Incontinence rose from 18% (3/17) at diagnosis to 87% (20/23) post-SRP; at longer-term follow-up (27.3 months), 79% (11/14) used ≥1 pad/day. Erectile dysfunction was present in 82% (14/17) at 22.0 months. Additional cancer-directed therapy was given in 50% (12/24). **Conclusions**: In this small, descriptive series, sRARP was feasible, but only 39% of patients were free of biochemical recurrence and functional burden was substantial. sRARP may represent a treatment option for carefully selected patients with recurrent localized prostate cancer; however, individualized counseling and further multicenter evaluation are essential.

## 1. Introduction

Prostate cancer (PCa) is one of the most commonly diagnosed cancers in men and a leading cause of cancer-related deaths, with an estimated 2.9 million new cases projected worldwide by 2040 [1]. The majority of patients with newly diagnosed PCa present with localized disease. Management is guided by disease risk-stratification and patient characteristics and may include radiotherapy or focal therapy [2]. Despite effective primary therapy, up to 30% of patients develop biochemical or local recurrence [3].

Salvage radical prostatectomy (SRP) is a potentially curative treatment option for patients with recurrent localized PCa [4]. Historically, SRP is associated with significant technical complexity, high complication rates, and poor functional outcomes and is, therefore, rarely performed [5]. In recent years, surgical advancements with the incorporation of robotic instrumentation and greater surgeon experience underscore the need to re-evaluate the use of SRP for recurrent localized PCa [6,7].

SRP carries a well-documented risk of urinary and erectile dysfunction beyond what is seen with primary radical prostatectomy; hence, functional status is a central consideration in patient counseling and treatment selection, alongside oncologic control [8,9]. Characterization of continence status and erectile function from initial diagnosis through recurrence and following SRP reflects the cumulative functional burden patients experience across the disease course [10].

Currently, much of the existing evidence is derived from systematic reviews or multi-institutional cohorts with heterogeneous surgical technique, follow-up, and outcome reporting [11]. In this study, we performed a retrospective review at a single institution over a two-decade period to better characterize contemporary clinical and functional outcomes of patients undergoing salvage robotic-assisted radical prostatectomy (sRARP) for recurrent localized PCa.

## 2. Materials and Methods

This is an IRB-approved retrospective chart review study under protocols IRB00109136 and IRB00149571 of all patients who underwent SRP at Atrium Health Wake Forest Baptist (AHWFB) Medical Center from 1 January 2004 to 30 June 2026. Patients were identified using the Informatics for Integrating Biology and the Bedside (i2b2) [12] clinical data repository platform by creating a temporal query of patients with a history of prostate cancer who subsequently underwent primary treatment with radiation therapy (external beam or brachytherapy) or focal therapy (high-intensity-focused ultrasound [HIFU] ablation, focal laser ablation, cryoablation, irreversible electroporation, or other focal modalities), followed by radical prostatectomy (open, laparoscopic, or robotic-assisted). Procedures were identified in i2b2 by their respective Current Procedural Terminology (CPT) codes. An additional search was completed in the institutional database using the SKAN Natural Language Processing (NLP) software (version 2.8.0) for keywords of “salvage radical prostatectomy” and related nomenclature variants. The sensitivity and specificity of these search strategies were not formally validated. All identified charts were reviewed individually to confirm performance of salvage radical prostatectomy and exclude non-salvage prostatectomy procedures and/or cases performed at outside hospitals.

A variety of clinicodemographic, perioperative, clinical, and functional data were collected through patient charts from the electronic medical record (EMR) at AHWFB. Clinicodemographic variables include patient age, race, comorbidities, body mass index (BMI), smoking history, age, and clinical Tumor, Node, Metastasis (TNM) staging at initial prostate cancer diagnosis, National Comprehensive Cancer Network (NCCN) risk stratification, and primary treatment option. Perioperative variables include years from primary therapy to recurrence, clinical TNM staging at recurrence, SRP surgical approach, surgery time, and any subsequent prostate cancer treatments. Functional outcomes were measured at initial diagnosis, at recurrence, and following salvage radical prostatectomy, and included the International Prostate Symptom Score (IPSS) [13], International Index of Erectile Function (IIEF) score using the 5-item version [14], presence of incontinence, presence of erectile dysfunction, and number of pads used per day.

Recurrence was defined by rising prostate-specific antigen (PSA) levels and/or confirmatory prostate biopsy. Additionally, post-surgical factors were identified including the presence of bladder neck contracture, final pathologic tumor stage and surgical margin status, performance of pelvic lymph node dissection and lymph node status, complications within 90 days of surgery graded according to the Clavien–Dindo classification system [15] and later complications (after 90 days of surgery), which were described with dates and not graded, length of hospital stay, duration of surgical (Jackson-Pratt) drain placement, receipt of additional cancer-directed therapy following SRP (e.g., androgen deprivation therapy [ADT] or radiation), postoperative PSA nadir and most recent PSA, and vital status at last follow-up.

Incontinence at initial diagnosis, at recurrence, and immediately following SRP was defined as any documented involuntary urinary leakage noted in the EMR. Continence at longer-term follow-up was defined more strictly by reported pad count (Continent: 0 pads/day; incontinent: ≥1 pad/day), assessed among the subset of patients with a quantified pad count recorded at that visit.

Biochemical recurrence-free survival (bRFS) was defined as freedom from both PSA persistence and biochemical recurrence. PSA persistence was a PSA that did not fall below 0.2 ng/mL after surgery; biochemical recurrence (BCR) was a PSA ≥ 0.2 ng/mL after an initial nadir below 0.2 ng/mL and documented as biochemical recurrence in the medical record, using the threshold recommended by the American Urological Association, although confirmatory values were not consistently available [16]. Recurrences documented in external records were included. Initiation of additional cancer-directed therapy (ADT or radiation) on or before the data cutoff of 7 September 2026 was also analyzed as an event. PSA nadir was the lowest PSA after surgery and before any additional therapy. bRFS was reported as a proportion of evaluable patients at last follow-up; because dated serial PSA values were not available for all patients, time-to-event analysis was not performed. Sensitivity analyses used PSA thresholds of 0.1 and 0.4 ng/mL. One patient with no documented PSA follow-up was excluded. This definition is distinct from the recurrence definition above, which pertains to disease recurrence following primary therapy that prompted SRP.

Complications within 90 days of surgery were graded by the most invasive intervention required to treat each complication, including interventions after 90 days for a complication that began within 90 days; events with the same causes were counted once, and each patient was assigned the highest grade. Complications after 90 days were recorded descriptively with dates, and those requiring a procedure were counted separately. Late events were identified by chart review through the last documented follow-up (up to 7 September 2026) and may be under-ascertained, particularly for events managed at outside institutions.

This study was designed as a descriptive characterization of a contemporary single-institution salvage radical prostatectomy cohort; no formal hypothesis testing or multivariable modeling was performed, given the limited sample size. Continuous variables are reported as median with interquartile range (IQR), given the small sample size and expected non-normal distribution of several variables; categorical variables are reported as a count over the number of patients with a non-missing value for that variable (n/N) and the corresponding percentage. Because data completeness varied across variables and across the three functional assessment timepoints (initial diagnosis, recurrence, and post-SRP), the denominator (N) is reported alongside each value rather than assuming complete data for the full cohort (*n* = 24). Functional outcomes are reported descriptively at each timepoint, without direct statistical comparison across timepoints, because assessment methods differed. Estimated blood loss recorded as an upper bound (for example, <50 mL) was analyzed at the bound value, which gives a conservative estimate. In this manuscript, SRP denotes salvage radical prostatectomy as a procedure, and sRARP denotes the robotic-assisted approach analyzed in this cohort (*n* = 24). Descriptive statistics were computed in Python (version 3.12.3; pandas) and organized into the summary tables presented as Table 1, Table 2, Table 3 and Table 4 using Microsoft Excel (Microsoft Corporation, Redmond, WA, USA).

Generative artificial intelligence (Claude Sonnet 5; Anthropic, San Francisco, CA, USA) was used during the preparation of this manuscript to (1) calculate the descriptive summary statistics and generate the formatted tables in Table 1, Table 2, Table 3 and Table 4 and (2) assist in drafting and revising portions of the manuscript text, including Section 2, Section 3 and Section 4, based on author-provided source data, prior manuscript drafts, and explicit author instructions. All artificial intelligence-assisted calculations, tables, and text were reviewed, verified against the source data, and edited by the author(s) prior to submission. The author(s) take full responsibility for the accuracy, integrity, and originality of the final published work.

## 3. Results

### 3.1. Patient Identification and Cohort

A total of 4002 unique records were identified through the combined i2b2 temporal queries and SKAN NLP keyword search. The majority were excluded on individual chart review, predominantly duplicate records returned by the multiple search strategies, as well as records not meeting inclusion criteria (non-SRP and/or procedures performed at an outside hospital). After the of 3972 records, a total of 30 patients underwent SRP at AHWFB Medical Center between 1 January 2004 and 30 June 2026, and met the inclusion criteria for this retrospective review. Of these 30 patients, 24 (80%) underwent a robotic-assisted approach, 5 (17%) underwent an open approach, and 1 (3%) had surgical approach not recorded. The analyses below focus on the robotic-assisted subset (*n* = 24) unless otherwise specified.

### 3.2. Baseline Clinicodemographic Characteristics (Table 1)

At initial diagnosis, baseline functional status was notable for a median IPSS of 3.0 (IQR 1.0–8.0; available in 13/24) and a median IIEF score of 17.0 (IQR 12.8–22.8; available in 6/24). Incontinence was present in 3/17 patients with data recorded (18%). Median PSA was 7.3 ng/mL (IQR 4.5–9.6; available in 21/24) and median Gleason score was 7 (IQR 7–7; available in 22/24). The clinical stage was most commonly cT1c (12/17 with staging recorded), and NCCN risk stratification was most often an intermediate risk (13/17 with a risk group recorded), including six favorable-intermediate, one unfavorable-intermediate, and six classified as intermediate risk without a favorable/unfavorable sub-designation documented in the chart; two patients each were low- and high-risk.

Primary treatment was radiation therapy in 13 patients (54%; 7 brachytherapy, 3 EBRT, 3 unspecified subtype), cryotherapy in 10 patients (42%), and HIFU in 1 patient (4%). Concurrent ADT was administered alongside primary therapy in two patients (8%).

**Table 1:** Baseline clinicodemographic characteristics of patients undergoing sRARP (*n* = 24).

Characteristic	n/N (%) or Median (IQR), *n*
**Demographics**	
Age at primary diagnosis, years	63.0 (57.0–66.0), *n* = 21
Age at SRP, years	68.1 (63.1–72.8), *n* = 24
Race	
White	20/24 (83%)
African American	4/24 (17%)
BMI, kg/m^2^	26.8 (24.9–28.9), *n* = 24
Normal weight	7/24 (29%)
Overweight	13/24 (54%)
Obesity Class I	3/24 (13%)
Obesity Class II	1/24 (4%)
Smoking	
Never	11/24 (46%)
Current	2/24 (8%)
Former	11/24 (46%)
**Comorbidities**	
Comorbidity	
Diabetes	5/24 (21%)
Hypertension	9/24 (38%)
Hyperlipidemia	6/24 (25%)
Coronary artery disease	1/24 (4%)
Chronic obstructive pulmonary disease	2/24 (8%)
Obstructive sleep apnea	7/24 (29%)
**Disease Characteristics at Initial Diagnosis**	
PSA at diagnosis, ng/mL	7.3 (4.5–9.6), *n* = 21
Gleason score at diagnosis	7.0 (7.0–7.0), *n* = 22
Clinical Stage	
cT1c	12/17 (71%)
cT2a	2/17 (12%)
cT2c	2/17 (12%)
cT3a	1/17 (6%)
NCCN Risk Stratification	
Low	2/17 (12%)
Intermediate, favorable	6/17 (35%)
Intermediate, unfavorable	1/17 (6%)
Intermediate, unspecified	6/17 (35%)
High	2/17 (12%)
**Primary Treatment**	
Radiation therapy	13/24 (54%)
Brachytherapy	7/13 (54%)
External beam radiation therapy	3/13 (23%)
Unspecified	3/13 (23%)
Cryotherapy	10/24 (42%)
HIFU	1/24 (4%)
Concurrent ADT with primary therapy	2/24 (8%)
**Functional Status at Initial Diagnosis**	
IPSS	3.0 (1.0–8.0), *n* = 13
IIEF	17.0 (12.8–22.8), *n* = 6
Incontinence	3/17 (18%)

Continuous variables reported as median (IQR), n = number of patients with a value recorded. Categorical variables reported as n/N (%), where N = number of patients with a value recorded for that field (denominator varies by row). Baseline incontinence was defined by documented urinary leakage in the medical record. Pad use was recorded for only 1 of 3 incontinent patients (0 pads/day); one patient was managed with a suprapubic tube. NCCN ‘Intermediate, unspecified’ = chart documented as intermediate risk without favorable/unfavorable sub-classification.

### 3.3. Recurrence and Perioperative Characteristics (Table 2)

Prior to SRP, a subset of patients received additional cancer-directed therapy following primary treatment, most commonly ADT (4/21 with data available, 19%), with radiation and cryotherapy each administered in one patient; these therapies were not mutually exclusive.

Median time from primary therapy to recurrence was 4.5 years (IQR 3.0–8.0), and median PSA at recurrence was 4.4 ng/mL (IQR 2.7–7.1). Median time from primary therapy to sRARP was 6.0 years (IQR 3.0–8.0). Functional status prior to SRP was notable for a median IPSS of 7.0 (IQR 2.0–13.5), median IIEF of 8.5 (IQR 5.0–13.2), and incontinence in 6/22 patients with data recorded (27%).

Median operative time was 173.0 min (IQR 152–196.0; *n* = 22) and median estimated blood loss was 50 mL (IQR 50.0–100.0). One patient (4%) experienced an intraoperative complication.

**Table 2:** Recurrence and perioperative characteristics of patients undergoing sRARP (*n* = 24).

Characteristic	n/N (%) or Median (IQR), *n*
**Additional Cancer-Control Therapy Prior to SRP**	
ADT	4/21 (19%)
Radiation	1/21 (5%)
Cryotherapy	1/21 (5%)
Years, primary therapy to additional therapy	2.0 (0.8–4.5), *n* = 4
**Recurrence Characteristics**	
Years, primary therapy to recurrence	4.5 (3.0–8.0), *n* = 22
PSA at recurrence, ng/mL	4.4 (2.7–7.1), *n* = 20
Gleason score at recurrence	7.0 (7.0–8.0), *n* = 15
Clinical Stage	
cT1c	1/5 (20%)
cT2	1/5 (20%)
cT2c	2/5 (40%)
cT3a	1/5 (20%)
**SRP Surgical Characteristics**	
Time, primary therapy to SRP, years	6.0 (3.0–8.0), *n* = 23
Operative time, minutes	173.0 (152.0–196.0), *n* = 22
Estimated blood loss, cc	50.0 (50.0–100.0), *n* = 22
Intraoperative complication	1/23 (4%)

Patients may appear in more than one row above if they received multiple concurrent salvage-directed therapies before SRP. Clinical T-stage at recurrence is reported among the small subset (*n* = 5) with staging documented at the time of recurrence.

### 3.4. Pathologic and Postoperative Outcomes (Table 3)

On final pathology, extraprostatic disease (pT3a or pT3b) was identified in 13/24 patients (54%), with pT2 disease in 7 (29%) and pT0 (no residual tumor) in 1. Surgical margins were positive in 10/23 patients with margin status recorded (43%). Pelvic lymph node dissection was performed in 23/24 patients (96%), with positive nodes identified in 4/23 patients who underwent dissection (17%).

Median length of hospital stay was 1.0 day (IQR 1.0–2.0). Bladder neck reconstruction was performed intraoperatively in four patients (17%). No patients died prior to discharge. One patient (4%) underwent concurrent ureterectomy with a Mitrofanoff appendiceal diversion at the time of SRP.

Within 90 days of surgery, 10 of 23 patients with data recorded (43%) had a complication: Clavien–Dindo grade I in 4, II in 3, IIIa in 2, and IIIb in 1. Three patients (13%) had a grade ≥ III complication: a pelvic collection requiring drain placement, suprapubic tube replacement for obstruction, and distal ureteral occlusion managed with percutaneous nephrostomy that ultimately required open ileal conduit diversion (graded by most invasive intervention). After 90 days, 7 of 23 patients (30%) had a complication, and 6 (26%) required a procedure, including urethral stricture or bladder neck contracture (*n* = 2), bladder stones with stoma dilation (*n* = 1), urinary diversion (*n* = 1), and image-guided drainage of an intra-abdominal collection (*n* = 2, on days 94 and 96, just outside the 90-day window). Bladder neck contracture developed in 4/22 patients with data recorded (18%). One patient (4%) subsequently required urinary diversion with an open ileal conduit, approximately 9 months after surgery.

Following SRP, 12 of 24 patients (50%) received additional cancer-directed therapy by the data cutoff, most commonly ADT (11/24, 46%) and, less frequently, radiation (4/24, 17%); some patients received both. PSA persistence occurred in 8 of 24 patients (33%). Median PSA nadir was 0.035 ng/mL (IQR 0.010–0.345; *n* = 22; unavailable in 2 patients). At a median PSA follow-up of 32.1 months (range 1.8–130.1; *n* = 19 with a dated last PSA), 9 of 23 evaluable patients (39%) were free of biochemical recurrence (bRFS). The other 14 had a treatment failure: PSA persistence in 8 and later biochemical recurrence in 6 (including one documented in outside records). Counting additional therapy as an event did not change this result, because every patient who received additional therapy already had a PSA ≥ 0.2 ng/mL at the time of treatment. bRFS was 30% (7/23) at a threshold of 0.1 ng/mL and 39% (9/23) at 0.4 ng/mL. Two of the nine patients free of biochemical recurrence had less than 3 months of PSA follow-up, and one patient with no documented PSA follow-up was excluded. As of the study’s chart-abstraction date (7 September 2026), 21/24 patients (88%) were alive; of the 3 deceased patients, none had a documented SRP-specific cause of death.

**Table 3:** Pathologic and postoperative outcomes of patients undergoing sRARP (*n* = 24).

Characteristic	n/N (%); Median (IQR) or Median (Range), *n*
**Pathologic Findings**	
Pathologic Stage	
pT0	1/24 (4%)
pT2	7/24 (29%)
pT2c	2/24 (8%)
pT3	1/24 (4%)
pT3a	8/24 (33%)
pT3b	5/24 (21%)
Margin status—Positive	10/23 (43%)
Margin status—Negative	13/23 (57%)
Lymph node dissection performed	23/24 (96%)
Lymph node positive	4/23 (17%)
Prostate size, post-SRP, g	36.0 (28.4–43.5), *n* = 23
**Postoperative Course**	
Length of hospital stay, days	1.0 (1.0–2.0), *n* = 23
Length of JP drain, days	1.0 (1.0–2.0), *n* = 20
Bladder neck reconstruction	4/23 (17%)
Died before discharge	0/24 (0%)
**Complications**	
Complication within 90 days	10/23 (43%)
Clavien–Dindo Grade	
Grade 1	4/10 (40%)
Grade 2	3/10 (30%)
Grade 3a	2/10 (20%)
Grade 3b	1/10 (10%)
Grade ≥ III, highest grade per patient	3/23 (13%)
Late complication requiring a procedure	6/23 (26%)
Bladder neck contracture	4/22 (18%)
Urinary diversion, later (ileal conduit)	1/24 (4%)
Urinary diversion, concurrent with SRP (Mitrofanoff)	1/24 (4%)
**Additional Cancer-Control Therapy After SRP**	
ADT	11/24 (46%)
Radiation	4/24 (17%)
**Oncologic/Survival Outcomes**	
PSA nadir post-SRP, ng/mL	0.035 (0.001–0.345), *n* = 22
PSA persistence (nadir ≥ 0.2 ng/mL)	8/24 (33%)
Median follow-up, months	32.1 (range 1.8–130.1), *n* = 19
Biochemical recurrence-free survival (bRFS)	9/23 (39%)
SRP-specific death (among deceased)	0/3 (0%)

Denominator (N) for each n/N (%) is the number of patients with a value recorded for that field, not always all 24 (e.g., pads/day is conditional on incontinence being present). Complications within 90 days were graded by Clavien–Dindo (highest grade per patient); late complications (after 90 days) are described, not graded. bRFS reflects freedom from PSA persistence and biochemical recurrence; counting additional therapy as an event gave the same result, ADT and radiation are not mutually exclusive: 12/24 (50%) received any additional therapy, and 3 received both.

### 3.5. Functional Outcomes over Time (Table 4)

Functional status was assessed at initial diagnosis, at the time of recurrence (immediately prior to SRP), and immediately following SRP. Median IPSS was 3.0 (IQR 1.0–8.0) at diagnosis, rising to 7.0 (IQR 2.0–13.5) before surgery and 11.0 (IQR 5.0–15.5) postoperatively, though data were available for a minority of patients at each timepoint (13, 15, and 8 of 24, respectively). Median IIEF was 17.0 (IQR 12.8–22.8) at diagnosis, 8.5 (IQR 5.0–13.2) before surgery, and 5.0 (IQR 5.0–5.0) postoperatively (6, 10, and 10 patients with values recorded, respectively), with the latter reflecting a floor effect consistent with anticipated post-prostatectomy erectile dysfunction. Incontinence increased from 3/17 (18%) at diagnosis to 6/22 (27%) at recurrence and 20/23 (87%) immediately after surgery.

At longer-term follow-up, distinct from the immediate postoperative assessment above, median follow-up specific to continence status was 27.3 months (range 2.1–69.0; *n* = 10); among the 14 patients with a quantified pad count at that follow-up, 11 (79%) reported using one or more pads per day. Median follow-up specific to erectile function was 22.0 months (range 0.4–125.3; *n* = 16); among the 17 patients with erectile function status documented, 14 (82%) reported erectile dysfunction.

Longitudinal functional data were incomplete. IPSS was available at all three timepoints in 1 of 24 patients and IIEF in 1 of 24, and no patients had IPSS, IIEF, and incontinence status all documented at every timepoint; incontinence status was documented at all three timepoints in 17 of 24 (71%). The reasons for missing functional data were not recorded. Because incontinence was documented from the medical record at diagnosis and recurrence but was also characterized by pad counts after SRP, and because IPSS and IIEF were captured opportunistically and were incomplete, values are reported descriptively at each timepoint without direct statistical comparison. Among the 17 patients with incontinence status documented at all three timepoints, incontinence was present in 3/17 (18%) at diagnosis, 4/17 (24%) at recurrence, and 14/17 (82%) following SRP. At last follow-up (continence status documented in 18 patients), 8 were incontinent, 5 were continent without a device, 3 were continent with an implanted device (1 artificial urinary sphincter and 2 slings), and 2 had urinary diversion; thus 3/24 patients (13%) underwent AUS or sling placement.

**Table 4:** Functional outcomes at initial diagnosis, prior to robotic-assisted SRP (at recurrence), following robotic-assisted SRP, and median follow-up for key functional outcomes (*n* = 24).

Measure	At Initial Diagnosis	Prior to SRP (at Recurrence)	Following SRP
IPSS, median (IQR)	3.0 (1.0–8.0), *n* = 13	7.0 (2.0–13.5), *n* = 15	11.0 (5.0–15.5), *n* = 8
IIEF, median (IQR)	17.0 (12.8–22.8), *n* = 6	8.5 (5.0–13.2), *n* = 10	5.0 (5.0–5.0), *n* = 10
Incontinence, *n* (%)	3/17 (18%)	6/22 (27%)	20/23 (87%)
Pads/day (if incontinent), median (IQR)	0.0 (0.0–0.0), *n* = 1	0.0 (0.0–0.5), *n* = 3	2.5 (1.0–4.8), *n* = 14
**Median Follow-Up, Months (Range)**			
Incontinence	27.3 (2.1–69.0), *n* = 10		
Erectile dysfunction	22.0 (0.4–125.3), *n* = 16		

Incontinence at diagnosis, at recurrence, and immediately following SRP reflects any documented involuntary urinary leakage noted in the chart. Incontinence at longer-term follow-up (≥1 pad/day) is defined more strictly by reported pad count, assessed only among patients with a quantified pad count recorded at that visit; these are not directly comparable measures. At initial diagnosis, pad use was recorded for only 1 of 3 incontinent patients. Follow-up duration reflects time from SRP to the most recent visit at which incontinence or erectile function, respectively, was documented, among patients with both the outcome and a corresponding follow-up date recorded.

## 4. Discussion

In this single-institution, two-decade retrospective review, 30 patients underwent SRP, of whom 24 (80%) underwent a robotic-assisted approach. We analyzed the robotic-assisted subset and found perioperative and pathologic outcomes that are broadly consistent with contemporary robotic-era series, a bRFS of 39% at a median follow-up of 32.1 months, and substantial functional burden (particularly urinary incontinence) across the disease and treatment course. These findings suggest that robotic-assisted SRP is technically feasible in carefully selected patients while underscoring the importance of longitudinal functional counseling rather than a single postoperative estimate of risk.

Median operative time was somewhat longer (173 vs. 145 min) and estimated blood loss was lower (50 vs. 100 mL) than in a recent 439-patient multicenter robotic salvage series [17]. Our positive margin rate (10/23, 43%) was higher than the pooled 26.1% [18] and the 29.7% in an 18-center series [19], but within the range reported in robotic series, from 39% in a 106-patient sRARP series [20], to as high as 65.6% across published reports [21]. Similarly, our rate of extraprostatic disease (pT3a/pT3b, 13/24, 54%) exceeded the roughly 45% reported in a comparable single-institution cohort spanning a similar time period, and our lymph node positivity rate (4/23, 17%) was higher than the 6% reported in that same series [22]. These differences may reflect our modest sample size, in which small absolute numbers translate to wide swings in percentage terms, and possibly a case mix enriched for more advanced or higher-risk recurrent disease at the time of referral for SRP; they should be interpreted with appropriate caution given the width of the confidence intervals implied by our denominators.

Within 90 days, 10 of 23 patients (43%) had any complication, 7 of them were grade I-II, and 3 (13%) had a Clavien–Dindo grade ≥ III complication. These rates are higher than the 8% overall 90-day complication rate in a 106-patient sRARP series [20] and the 6.6% pooled high-grade rate [18]; how minor events such as emergency department visits for catheter problems are captured and graded likely contributes to the rates, as do our small sample and referral-center case mix. Late complications (after 90 days) occurred in 7 of 23 patients (30%), and 6 (26%) required a procedure; two image-guided drainages fell just outside the 90-day window (days 94 and 96), and extending the window to 100 days would raise the grade ≥ III rate to about 5/23 (22%). Notably, 1 patient (4%) required later urinary diversion with an ileal conduit for ureteral stricture and urine leak, and another underwent a planned Mitrofanoff appendiceal diversion at the time of salvage surgery. While uncommon, these findings underscore that the most severe morbidity after SRP can extend beyond incontinence and erectile dysfunction to altered urinary tract anatomy, and that perioperative grading alone underestimates the total morbidity of salvage surgery; this should be discussed with patients as part of comprehensive preoperative counseling.

At a median PSA follow-up of 32.1 months, 39% of our robotic cohort (9/23) were free of biochemical recurrence, and the result was similar when PSA thresholds of 0.1 and 0.4 ng/mL were applied (30% and 39%). Reported oncologic outcomes after salvage radical prostatectomy vary with the definition of recurrence and the length of follow-up [16]. In an 18-center series of 414 patients, 5-year BCR-free survival was 56.7%, and pathologic stage ≥ pT3b and higher grade independently predicted recurrence [19]. A systematic review of 20 studies (4175 men) reported 10-year metastasis-free survival of 72–77% and 5-year cancer-specific survival of 86.6–97.7% [18], and a systematic review of robotic salvage series reported BCR rates of 0–57% [21]. Our bRFS is lower than most published estimates. This may reflect our stricter definition, which counted PSA persistence and additional therapy as failure, an adverse pathologic profile (54% extraprostatic disease, 43% positive margins, and 17% node-positive disease, compared with pooled positive margin rates of 26.1% [18] and 29.7% [19]), and our small sample. A third of patients (8/24) had PSA persistence, meaning the PSA never fell below 0.2 ng/mL, which suggests residual disease outside the surgical field at the time of operation; persistence occurred after both radiation (five patients) and cryotherapy (three patients) as primary therapy. These results do not support the view that sRARP alone provides durable disease control in patients with unfavorable pathology; rather, a substantial proportion of patients will need additional treatment, which should be part of preoperative counseling. Descriptively, a bRFS was 4/12 after primary radiation, 5/10 after cryotherapy, and 0/1 after HIFU; the numbers were too small for formal comparison. bRFS was reported as a proportion and not a time-to-event estimate, so the timing of recurrence could not be characterized.

Salvage radical prostatectomy is one of several options for locally recurrent disease after radiotherapy. A meta-analysis of 150 studies found similar adjusted 5-year relapse-free survival across salvage radical prostatectomy, cryotherapy, HIFU, stereotactic body radiotherapy (SBRT), and brachytherapy (50–60%), but higher severe genitourinary toxicity after radical prostatectomy (20%) than after SBRT (5.6%), high-dose-rate brachytherapy (9.6%), or low-dose-rate brachytherapy (9.1%) [23]. This is consistent with the functional burden we observed and indicates that radical prostatectomy should be reserved for patients in whom the chance of cure justifies that burden. Our single-arm cohort cannot assess comparative effectiveness. Adherence to the European Association of Urology criteria for salvage radical prostatectomy has been associated with a lower risk of BCR and metastasis after surgery [24].

Patient selection is central to outcomes after salvage radical prostatectomy. Relevant factors include life expectancy, baseline urinary and erectile function, tumor location and extent, and patient preference; these were not systematically recorded in our dataset. In a prospective trial of 635 patients with biochemical recurrence after prostatectomy or radiotherapy, prostate-specific membrane antigen (PSMA) positron emission tomography (PET) localized recurrence in 75% with detection rising from 38% at PSA < 0.5 ng/mL to 97% at ≥5 ng/mL [25]. A consensus system, Prostate Imaging for Recurrence Reporting (PI-RR), now standardizes magnetic resonance imaging (MRI) reporting of local recurrence [26], and PSMA PET/computed tomography has been reported to help select patients for salvage radical prostatectomy after radiotherapy [27]. The present utilization of multiparametric MRI and PSMA PET may improve patient selection, although our study could not test this.

Our continence outcomes were less favorable than some published series. At ≥2 years, 50% of patients in a 106-patient sRARP series were fully continent and 33% socially continent [20], and a systematic review reported continence preserved in 40.4% [18]; in our cohort, 79% of patients with a quantified pad count (11/14) used ≥1 pad/day at a median of 27.3 months. Definitions of continence differ across studies, over half of our patients had prior radiation (13/24), 18% developed bladder neck contracture, and opportunistically captured data may over-represent symptomatic patients. Technical modifications that aim to improve early continence recovery after primary robotic prostatectomy [28] were not captured in our dataset. In a multicenter comparison, robotic salvage prostatectomy was associated with fewer anastomotic strictures, less blood loss, a shorter hospital stay, and better continence than open surgery [29].

The most notable finding in our cohort was the magnitude of functional burden observed, both across the three assessed timepoints and at longer-term follow-up. Incontinence rose from 18% (3/17) at initial diagnosis to 27% (6/22) at recurrence to 87% (20/23) immediately following SRP. At a median continence-specific follow-up of 27.3 months, 79% of patients with a quantified pad count continued to report using 1 or more pads per day. Erectile dysfunction followed a similar pattern, present in 82% of patients at a median follow-up of 22.0 months, consistent with the near-universal erectile dysfunction described after SRP in prior literature [8,9]. As in our overall cohort, functional data were available for a minority of patients at each timepoint, and if patients with worse functional outcomes were more likely to have that outcome documented (e.g., through a subsequent urology visit for incontinence management), our reported rates may be inflated by informative missingness, a limitation inherent to retrospective chart review rather than prospectively administered, protocol-driven questionnaires. Oncologic control alone does not capture the benefit of salvage surgery; patient-reported outcomes and decision regret are essential to evaluating salvage strategies [8]. Because baseline incontinence relied on chart documentation while postoperative continence was also characterized by pad counts, we avoid direct statistical comparison across timepoints.

This study benefits from a two-decade single-institution experience and, notably, granular functional data captured at three distinct timepoints in addition to longer-term, outcome-specific follow-up for continence, erectile function, and biochemical recurrence, rather than the single postoperative snapshot typical of much of the existing literature.

Several limitations warrant consideration. First, this is a retrospective, single-institution study of a small cohort (*n* = 24 robotic-assisted cases); the study period (2004–2026) spans substantial changes in robotic platforms, surgeon experience, patient selection, staging imaging (including multiparametric MRI and PSMA PET), approaches to recurrent assessment, and perioperative care, and we could not evaluate how these changes influenced outcomes. Restricting to the robotic subset further limits precision and widens the confidence interval around every reported rate. Second, these results reflect a highly selected population treated at a tertiary referral center and should not be interpreted as evidence supporting routine adoption of sRARP in all patients with recurrent prostate cancer; the factors that guided patient selection, such as life expectancy baseline urinary and erectile function, tumor localization, imaging findings, and patient preference, were not systematically recorded. Third, the cohort was heterogeneous with respect to primary therapy (radiation, cryotherapy, and HIFU), and the sample was too small for stratified analysis. Fourth, missing data were substantial and uneven across variables and timepoints, particularly for patient-reported functional measures (IPSS, IIEF), which were not administered on a fixed protocol schedule but captured opportunistically through the electronic medical record; the reasons for missing data were not recorded, and this may introduce non-random missingness bias as discussed above. Fifth, bRFS was reported as a proportion and not a time-to-event estimate because dated serial PSA values and confirmatory values were not available for all patients; some events were identified from outside records, and two patients free of biochemical recurrence had less than 3 months of PSA follow-up. Sixth, complications within 90 days were graded, whereas later complications were described without grades; follow-up duration varied, additional therapies may have contributed to some late events, and late events may be under-ascertained, particularly those managed at outside institutions. Seventh, cases were identified through i2b2 queries and an NLP keyword search whose sensitivity and specificity were not validated, so some eligible cases may have been missed, such as cases not labeled as salvage, or care at external hospitals. Eighth, operative details such as nerve-sparing status, lymph node yield, and intraoperative findings were not consistently captured. Additionally, the relatively short median follow-up (32.1 months) limits our ability to capture late biochemical recurrences, which are well described in prostate cancer even beyond 5 years. Finally, as a single tertiary referral center, our case mix and surgical technique may not be generalizable to other practice settings.

## 5. Conclusions

This contemporary, single-institution, descriptive cohort suggests that sRARP may represent a treatment option for carefully, selected patients with recurrent localized prostate cancer. The present utilization of multiparametric prostate magnetic resonance imaging and prostate-specific membrane antigen positron emission tomography scan may improve patient selection. However, only 39% of patients were free of biochemical recurrence at a median follow-up of 32.1 months, and the substantial and progressive functional burden observed, particularly urinary incontinence, together with severe complications including later urinary diversion in one patient, highlights the importance of individualized, longitudinal preoperative counseling. Larger, prospective, multi-institutional studies with standardized, protocol-driven functional assessment and confirmed biochemical endpoints are needed to define predictors of outcomes and to refine patient selection for this procedure.

## Data Availability

The data in this study is not publicly available due to patient privacy but is available in de-identified format upon reasonable request to the corresponding authors.

## Abbreviations

The following abbreviations are used in this manuscript:

PCa	Prostate Cancer
SRP	Salvage Radical Prostatectomy
sRARP	Salvage Robotic-Assisted Radical Prostatectomy
AHWFB	Atrium Health Wake Forest Baptist
i2b2	Informatics for Integrating Biology and the Bedside
HIFU	High-Intensity-Focused Ultrasound
CPT	Current Procedural Terminology
NLP	Natural Language Processing
EMR	Electronic Medical Record
BMI	Body Mass Index
TNM	Tumor, Node, Metastasis
NCCN	National Comprehensive Cancer Network
IPSS	International Prostate Symptom Score
IIEF	International Index of Erectile Function
PSA	Prostate-Specific Antigen
JP	Jackson-Pratt
bRFS	Biochemical Recurrence-Free Survival
BCR	Biochemical Recurrence
IQR	Interquartile Range
EBRT	External Beam Radiation Therapy
ADT	Androgen Deprivation Therapy
AUS	Artificial Urinary Sphincter
SBRT	Stereotactic Body Radiotherapy
PSMA	Prostate-Specific Membrane Antigen
PET	Positron Emission Tomography
MRI	Magnetic Resonance Imaging
